# Lesion-site-dependent responses to therapy after aphasic stroke

**DOI:** 10.1136/jnnp-2017-317446

**Published:** 2018-04-17

**Authors:** Oscar M Aguilar, Sheila J Kerry, Yean-Hoon Ong, Martina F Callaghan, Jennifer Crinion, Zoe Victoria Joan Woodhead, Cathy J Price, Alexander P Leff, Thomas M H Hope

**Affiliations:** 1 Department of Brain Repair and Rehabilitation, University College London, London, UK; 2 Wellcome Centre for Human Neuroimaging, University College London, London, UK; 3 Facultad de Psicologia, Pontificia Universidad Javeriana, Bogota, Colombia; 4 Institute of Cognitive Neuroscience, University College London, London, UK; 5 Department of Experimental Psychology, University of Oxford, Oxford, UK

**Keywords:** speech therapy, stroke, rehabilitation, image analysis

## Introduction

Stroke survivors with language difficulties (aphasia) vary: some recover quickly while others suffer long-term impairments, and different patients respond differently to the same speech and language therapies.^1^ In recent years, we and others have shown that much of the variability in language outcomes after stroke can be explained by reference to the details of the brain damage that individual patients have suffered.^2^ Here, we show that this same information can be used to predict responses of patients who had stroke to treatment.

## Methods

Detailed methods are provided in online supplementary material: here, we summarise the key points. Our focus is on a novel treatment for Central Alexia (CA): an acquired reading disorder in the context of a general language impairment (aphasia). Patients with CA are slow to read, make frequent errors and have additional problems with spoken and written language. Our intervention is a computerised therapy embodied in an application called ‘iReadMore’, which uses multimodal cueing and massed practice to improve patients’ single-word reading skills.^3^

10.1136/jnnp-2017-317446.supp1 Supplementary file 1

Our study included 23 participants with CA after left hemisphere stroke (see online supplementary table), recruited through both the PLORAS project^2^ and the outpatient speech and language therapy services at the National Hospital for Neurology and Neurosurgery, University College London Hospitals. Before the treatment began, each participant’s cognitive skills were assessed with an extensive protocol including linguistic and non-linguistic tests, yielding a total of 28 pretreatment behavioural variables per patient. We also acquired structural MRI from each patient, extracting lesion images using the Automatic Lesion Identification toolbox.^4^ The outputs (binary lesion images) were encoded as the proportion that each lesion destroyed, of a series of 69 anatomically defined regions of interest. With four demographic variables (age at therapy onset, time since stroke, sex and lesion volume), we had a total of 101 pretreatment variables to consider; our dependent variable was absolute change in the participants’ single-word reading skills.

We ran two analyses with these data. Our first, ‘explanatory’ (in-sample) analysis quantified the relative utility of: (1) behavioural and demographic data and (2) lesion location data, in explaining individual patients’ responses to treatment (ie, improvement in single-word reading accuracy). We did this by fitting linear models using each set of variables, both separately and in combination, using the Automatic Linear Modelling (ALM) facility distributed with the SPSS software package. Our second, ‘predictive’ (out-of-sample) analysis asked whether the pretreatment data could be used to predict responses in new patients. We did this by embedding the ALM process within a cross-validation process.

## Results

### Analysis 1: explanatory (in-sample) analysis

The Aikaike Information Criterion (AIC) for the model derived from behavioural and demographics data alone was higher (worse) than that selected from lesion data alone (81.75 vs 69.35), and the model selected from all of the data together was better still (AIC=47.98). Bayes factors corresponding to these differences were 493 (behavioural and demographics vs lesions) and 43 696, respectively (all data together vs lesions alone): that is, very strong evidence both (1) that lesion data explain more of the variance in the patients’ treatment responses than the behavioural and demographic data alone and (2) that the combination of the data yields a better explanation than either set alone. Only the ‘neuroimaging only’ and ‘combined data’ models were significant, as assessed relative to null distributions of regression coefficients derived when the same models were regressed against 1000 permutations of the treatment responses (ie, actual R^2^>95% of null statistics).

**Figure 1:**
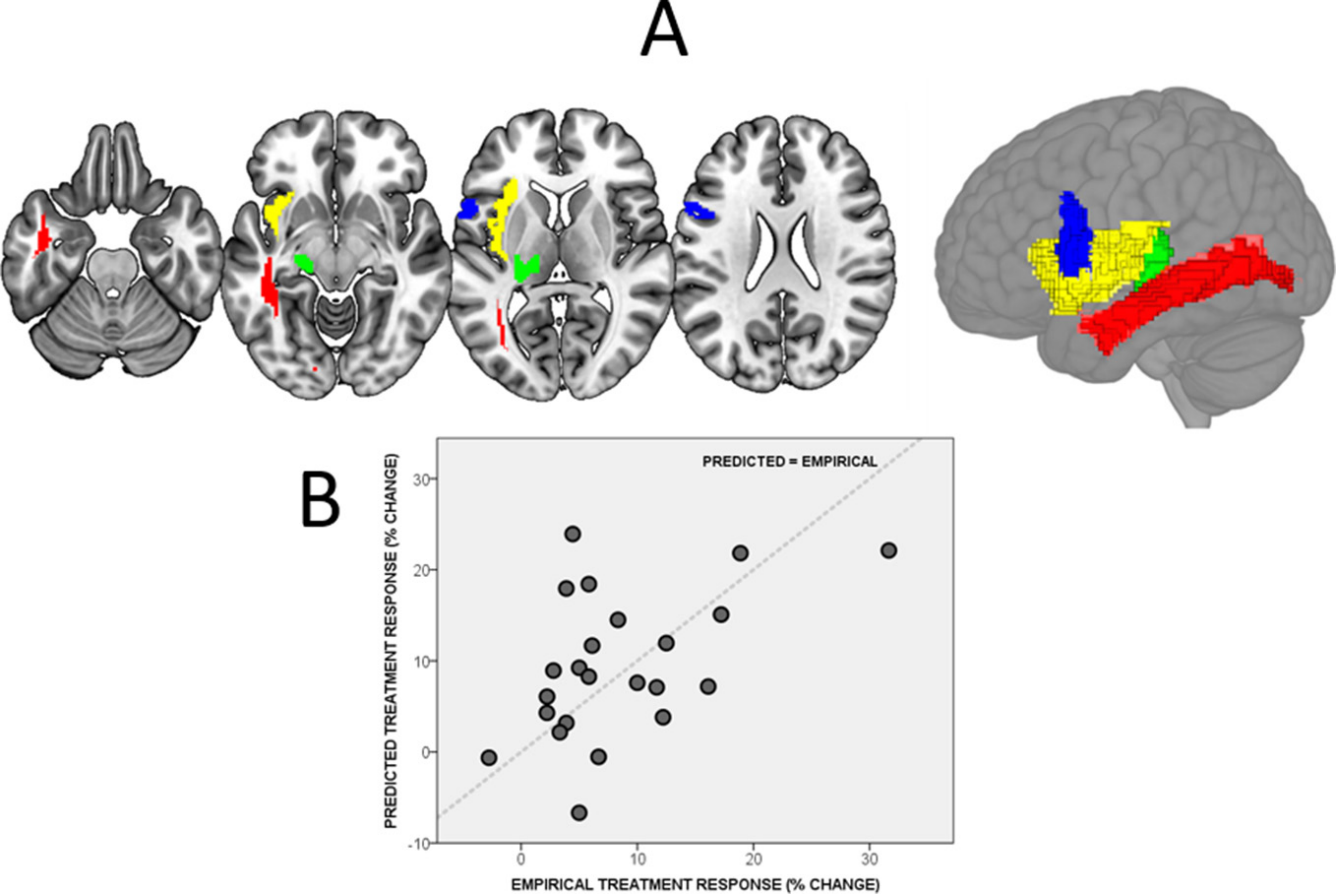
(A) The brain regions implicated in our combined model, displayed both on axial slices of the brain (left) at Z = −24, –8, 7 and 23 and on a rendered whole brain (right). The regions are: (a) the white matter connecting the thalamus to the parietal cortex (green); (b) the inferior longitudinal fasciculus (red); (c) Broca’s area 44 (blue) and (d) the left insula (yellow). (B) A scatter plot of predicted versus empirical treatment responses from our out-of-sample analysis. The dashed line is at y=x: perfect predictions would fall along this line.

The best, explanatory model that we found included: (1) age at therapy onset; (2) accuracy in the Neale reading test; (3) comprehension in the Neale reading test; (4) accuracy in the written semantic matching task; (5) damage to the white matter connecting the thalamus to the parietal cortex and damage to left (6) Broca’s area; (7) insula and (8) inferior longitudinal fasciculus (adjusted R^2^=0.94). Figure 1A displays the brain regions from this model.

### Analysis 2: predictive (out-of-sample) analysis

Predicted treatment responses from the cross-validation analysis, using the combined demographic, behavioural and lesion location data, were significantly correlated with the patients’ empirical treatment responses (r=0.48, 95% CI 0.08, to 0.75, p=0.02); see figure 1B. Predictions driven by models using either demographic and behavioural data or lesion location data, separately, were not significantly correlated with the empirical treatment responses (both p>0.1).

## Discussion

Past studies have suggested that responses to treatment for word-finding deficits (anomia) after stroke may be related to pretreatment behavioural skills^5^ or lesions.^6 7^ Our results are consistent with that work and also extend it by: (1) quantifying the relative value of pretreatment structural MRI versus demographic and behavioural data, in explaining treatment responses and (2) demonstrating that, in combination (though not separately), these pretreatment data can be used to predict new patients’ responses to treatment.

We have only considered a single therapy (iReadMore), focused on a specific aphasic deficit (CA), in a study with specific inclusion criteria at both the lesion level and the behavioural level (see online supplementary material). And though large by the standards of other similar therapy studies, our sample is still too small either to properly constrain multivariable models or to measure their predictive power precisely. So our results are necessarily preliminary.

However, they are also plausible. Cognitive neuroanatomy is specialised: the lesion damage that patients have suffered should determine both their initial symptoms and their likely potential to recover. Our best model of those treatment responses is also reasonable. Initial reading accuracy is naturally relevant to patients’ likely gains after reading therapy, and preserved semantic skills have been associated with recovery from aphasia.^8^ The inferior longitudinal fasciculus, the insula and Broca’s area have all been associated with reading in neuroimaging experiments,^9–12^ and it is at least plausible that older patients responded less well to our treatment. Finally, our predictive analysis should penalise small samples, because these will tend to maximise the variability between folds of the cross-validation process. Indeed, our results demonstrate the gulf that separates prediction from in-sample analysis: we could ‘explain’ 94% of the variance in those treatment responses, but predict only 23%. The out-of-sample effect size should grow with increasing sample size, but the in-sample effect size is clearly inflated.

In any case, we hope that these results will encourage further attempts to characterise lesion-site-dependent treatment effects and to distinguish predictable variance from noise in this area—and that further results like this will drive the development of a more personalised medicine for stroke survivors with aphasia.
