# Supplementary material for: Lesion-site-dependent responses to therapy after aphasic stroke
Source: J Neurol Neurosurg Psychiatry. 2018 Apr 17;89(12):1352–4. doi: 10.1136/jnnp-2017-317446 (PMC6288693; doi:10.1136/jnnp-2017-317446)
Supplement: Supplementary file 1 [file jnnp-2017-317446supp001.pdf]

Supplementary material

1. Testing of reading accuracy:

Words with high written frequency (SUBTLEXWF > 50) were selected from the SUBTLEX database [1]. High frequency words were chosen to maximise the ecological utility of the therapy. All words were three to six letters long so that they could easily be read in one fixation. Hyphenated or punctuated words were excluded, and an effort was made to avoid regular morphological variants of the same word (e.g. eat, eaten, eating). Words of all classes (nouns, verbs, function, etc.) were included, including both high and low imageability words.

Three matched lists of 180 words were created (A, B and C). For each word on list A there was a corresponding word on lists B and C closely matched for letter length, syllable length, written frequency and imageability. Additionally, the 50 highest frequency words (mostly function words) were selected as a separate list of 'Core' words.

All 590 words were tested at baseline (split across T1 and T2 sessions). Results from this full corpus of testing items were used to establish the participants' profiles of reading impairment. Based on each participant's baseline performance, a customised set of 150 matched words from each of the A, B and C lists were selected to use in training and subsequent assessments. This ensured the A, B and C lists selected for that patient were matched for baseline reading performance (word reading accuracy and RT). Furthermore the lists remained matched for psycholinguistic variables.

The A, B and C Word-Lists were assigned to be either trained in Block1 (data used in this paper), trained in Block2 or not to be trained (untrained words). List allocations were counterbalanced between participants. All 50 Core words were trained in both Block1 and Block2 due to their high utility.

From the customised 150-item A, B and C word lists, a subset of 90 items from each list were selected for use in all subsequent assessment time-points (T3-T6). These 90-item testing lists were matched for baseline performance and psycholinguistic variables. Importantly, the overall accuracy of the word lists selected for testing was matched to Baseline reading accuracy to avoid the risk of regression to the mean at future time-points.

2. Central Alexia and its treatment

All participants in this study were assessed as suffering from Central Alexia (CA). CA is an acquired reading disorder in the context of more generally impaired language (aphasia)[2]. Patients with CA are slow to read, make frequent errors and have additional problems with spoken language. Following the Connectionist 'triangle model' of reading, variants of CA are caused by damage to one of two routes linking orthography (how a word looks) to phonology (how it sounds): a direct route and an indirect route mediated by semantic processes (what the word means)[3]. Damage to the direct route primarily affects pseudoword reading, and is commonly called 'phonological dyslexia', though severe cases (called 'deep dyslexia') may involve semantic errors as well[4]. By contrast, damage to the semantically mediated route impairs irregular word reading, and is called 'surface dyslexia'[5].

iReadMore, the treatment in this study, is an application installed on the participants' computers, for use at home[6]. The application works by involving patients in massed practice of single-word reading, supported by multi-modal cueing (e.g. the word 'CAR' is presented along with a picture of a car, and the sound of a person saying the word). Participants used the application for four weeks at home in each treatment block: our main analysis is concerned with the first of those treatment blocks, with treatment response measured as the absolute change in participants' single-word reading accuracy on words encountered during the treatment (i.e. trained items). Absolute change in reading accuracy is expressed as a percentage of the total number of items in the assessment that participants could read correctly. Although accuracy was calculated separately for both trained and untrained words, in this study we only reported reading accuracy change on trained items (i.e. where we expected to see the response to treatment expressed). Change was chosen as the dependent variable because the sample of patients recruited in this study had variable severity of reading impairments at baseline.

3. **Supplementary table:** Demographic and clinical information on each patient.

Patient ID	Age (years)	Sex M/F	Time post-stroke (months)	Lesion Volume (cm³)	Handedness R/L	CA subtype	Baseline reading accuracy (%)	Pre-treatment (T3) reading accuracy (%)	Post-treatment (T4) reading accuracy	**Reading absolute change (%)**
**P01**	44	M	94	240.9	R	D	58.4	31.7	63.3	**31.7**
**P04**	52	M	66	122.7	R	P	71.1	65.6	84.4	**18.9**
**P02**	50	M	82	304.5	R	D	40.3	26.1	43.3	**17.2**
**P21**	58	F	41	297.7	R	P	59.5	67.2	83.3	**16.1**
**P08**	67	M	107	11.7	R	D	12.5	18.3	30.8	**12.5**
**P22**	42	M	13	43.7	L	P	74.9	76.7	88.9	**12.2**
**P09**	43	F	55	399.2	R	D	58.2	75.0	86.7	**11.7**
**P17**	60	M	16	102.6	R	D	28.1	34.2	44.2	**10.0**
**P05**	56	F	93	149.8	R	S	63.8	52.2	60.6	**8.3**
**P23**	26	F	81	161.9	R	D	75.5	72.2	78.9	**6.7**
**P15**	54	M	39	189.7	R	P	47.3	62.2	68.3	**6.1**
**P16**	73	M	158	205.2	R	D	20.0	20.0	25.8	**5.8**
**P20**	72	M	101	243.3	R	D	13.4	20.0	25.8	**5.8**
**P10**	61	M	19	195.6	R	D	3.4	11.7	16.7	**5.0**
**P19**	50	F	72	141.3	R	P	35.9	41.1	46.1	**5.0**
**P13**	54	M	24	149.3	R	P	91.5	90.0	94.4	**4.4**
**P06**	55	F	75	151.2	R	P	91.9	96.1	100.0	**3.9**
**P11**	52	M	12	31.2	R	P	96.3	88.9	92.8	**3.9**
**P14**	56	M	23	45.1	R	P	80.4	86.1	89.4	**3.3**
**P07**	33	F	59	181	R	P	90.1	93.3	96.1	**2.8**
**P12**	50	F	14	59.4	R	P	90.6	94.4	96.7	**2.2**
**P18**	78	M	22	128.5	L	P	75.4	77.8	80.0	**2.2**
**P03**	64	M	25	102.7	R	P	96.7	99.4	96.7	**-2.8**

R= right; L= left; CA= central alexia; P= phonological alexia; S= surface alexia; D= deep alexia. In bold, absolute change in reading accuracy after treatment (dependent variable).

4. Supplementary figure: study design.

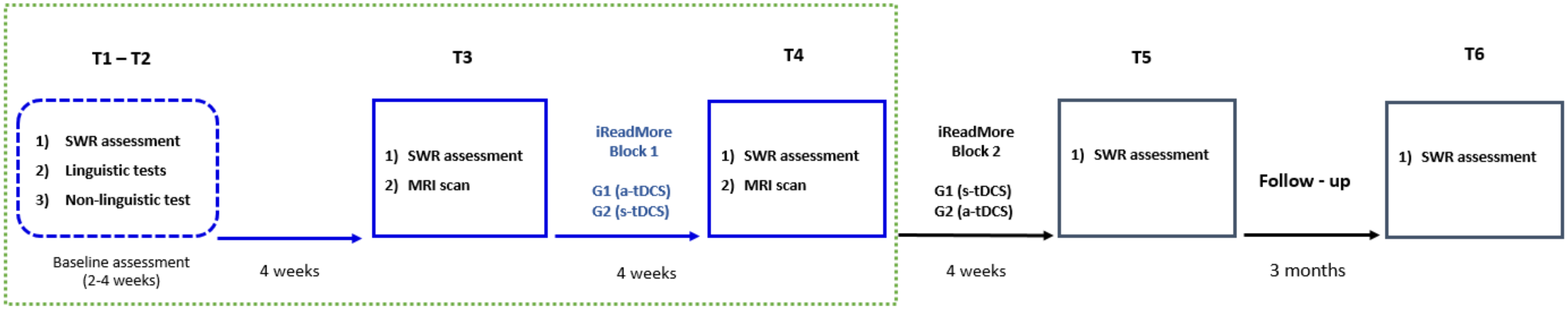

The current study (T1 - T4, within the green dotted line) is a subset of a larger longitudinal study (T1 – T6). It involved baseline behavioural testing (T1 – T2), and pre-treatment and post-treatment (T3-T4) reading testing and MRI scan. In the first block of therapy, participants received behavioural training (iReadMore) and tDCS for 4 weeks. For tDCS, patients were randomly allocated in two groups to receive real or sham tDCS. The larger study (outside green dotted line) included a second block of therapy and tDCS. In this block, for tDCS patients re ceived the opposite stimulation to the received in block 1. SWR= single-word reading task; MRI= structural magnetic resonance imaging; G= group; tDCS: transcranial direct current stimulation; a-tDCS: anodal tDCS; s-tDCS: sham tDCS

5. MRI data acquisition and pre-processing

At T3 each patient underwent a quantitative multi-parameter mapping protocol at 3T (Magnetom TIM Trio, Siemens Healthcare, Erlangen, Germany) using a standard 32 channel head coil for signal reception. The sequence parameters were as described in [7] with the exceptions that the FLASH data were acquired with 1mm isotropic resolution and a field of view of 256mm (HF) x 240mm (AP, 44 reference lines) x 176mm (RL, 40 reference lines) [8]. Using the data from this protocol, maps of magnetisation transfer saturation were calculated as described in [9]. These maps were subsequently used as input to a unified segmentation algorithm [10], which was optimized for use in patients with focal brain lesions [11]. The segmentation routine resulted in a binary lesion image for each patient, in standard MNI space. Lesion volume was calculated from that image.

We also encoded the lesion images by lesion load (percentage of damage) in a series of anatomically defined regions of the brain: 0% if the region was completely preserved by a patient's lesion(s), rising to 100% when the region was completely destroyed. We extracted 398 regions for this process, from a series of publically available atlases of grey and white matter regions [12-15]. The aim here was to cover the whole brain (i.e. GM and WM), encoding patients' lesions with minimal a priori assumptions concerning what lesion locations might be most relevant to the patients' treatment responses. We further reduced the resulting data by including only those (69) regions where at least 10 patients' lesions had destroyed at least 10% of the region; this was deemed necessary to exclude regions for which reliable correlations between lesion load and treatment response could not be measured.

The multi-parameter mapping protocol was preferred because we hoped to use it to identify longitudinal structural changes associated with therapy, which are more mechanistically interpretable than those that can be derived from traditional grey and white matter segmented images (this is in preparation). In this sense, the use of this protocol is not strictly required for this study. But the MT images derived from this process are also more than suitable for lesion segmentation, using the Automatic Lesion Identification toolbox, and the results were assessed by eye, by a neurologist (APL) to confirm this.

6. Baseline behavioural assessment - Instruments

Patients' baseline behavioural abilities (T1 – T2) were assessed with an extensive protocol including linguistic and non-linguistic tests, yielding a total of 36 pre-treatment behavioural variables for each patient.

Linguistic tasks:

1) Single-word reading (SWR): this task was designed to assess singl e-words reading at each time point. All words from the A, B, C and Core training lists described above (590 words in total) were tested at the baseline (T1 and T2). Words were presented in a random order using E-prime software. Words were displayed in black, lower case, size 36 Arial font on a grey background. Participants were instructed to read the words aloud into a voice-key microphone as fast and accurately as they could. Participants were given up to four seconds to read the word: responses after this time were scored as incorrect. Reaction time was recorded by the voice-key. The outcome measures were percentage accuracy and mean reaction times.

2) Pseudoword reading: 20 pseudowords were generated using Wuggy software [16]. Items were between three and six letters in length and were made up of plausible letter combinations. Pseudowords were presented in using E-prime and displayed in black, lower case, size 36, Arial font on a grey background. The outcome measures were percentage accuracy and mean reaction times (RT).

3) Written semantic matching: three words were displayed on the screen in each trial using E-prime. One word at the top of the screen was the 'probe', and the task was to decide which of the two words displayed below it were most semantically related to the probe. Participants were instructed to read the three words silently to identify the target as fast as they could with a button press. Accuracy and reaction times were recorded.

4) Written sentence to picture matching (sentence reading): This task was created to assess silent reading for meaning. It consisted of 60 trials, presented in E-prime, in which patients silently read a sentence of between five and eight words. They were requested to read each sentence as fast as they could. A picture was then displayed on screen and the participant responded verbally whether the picture was congruent with the sentence or not (50% were congruent). Outcome measures were percentage accuracy on the picture decision task and sentence reading speed in words per minute (WPM).

5) Neale Analysis of Reading Ability test: In this task, participants read aloud two simple passages of standardized prose [17]. If a participant is unable to read a word within four seconds, it was provided by the experimenter. Comprehension questions were asked

after each text was read. The test has three components: reading accuracy, reading speed and reading comprehension.

6) Communication Disability Profile [18]: this is a patient-reported questionnaire for aphasic patients focused on activities of daily life. Only the reading section was tested before therapy started to provide a self-report measure of reading ability. It consists of four questions asking for silent reading of: 1) a single words; 2) a headline; 3) a whole story in a paper; and, 4) a letter. Outcome measure: overall score (maximum score = 16).

7) Naming objects and naming actions of the Comprehensive Aphasia Test [19]: these tasks include respectively 24 and 5 black and white drawn pictures. Participants were instructed to retrieve the name of the picture or find the word to describe the action. The outcome measure was total score from both tests (maximum score = 58).

8) Digit span of the Wechsler Adult Intelligence Scale IV [20]. This task was used to test attentional span and verbal working memory. This subtest involves repetition of number strings forward and backward. The outcome measure was total score from both tests transformed into a scaled score.

9) Auditory discrimination task [21]: this task assesses acoustic-phonological perception. It was used to provide a measure of the participant's ability to discriminate phonemes. The task consisted of three auditory non-words displayed in E-prime. In each trial the first or last stimulus is identical to the stimulus in the middle. Participants were instructed to identify the odd-one-out by button press. The outcome measure was the score, calculated by averaging the last 4 levels accomplished.

Non-Linguistic tasks

10) Pyramids and Palm Trees (pictorial version) [22]: this task was was used to test access to visual semantic information .The task consists of 52 trials. In each trial three pictures are shown (a probe picture and two pictures below). One picture is a semantically-related target and the other an unrelated distractor. Participants were asked to select the pictures semantically-related to the target picture. The outcome measure was total score (maximum score = 52).

11) Subtests 1 and 2 of the Cattell Culture Fair test [23]: these subtests were used to examine fluid intelligence and reasoning. Subtest 1 is a pattern completion task (12 trials). Participants had to choose which drawing out of five options, completed the pattern. Subtest 2 is an odd-one-out task (14 trials). Five black and white drawings were presented

and participants had to identify the odd-one-out. The outcome measure was total score from both subtests (maximum score = 26).

12) Two-Armed Bandit Task: this task is a modified version of the decision making task used to assess environmental and reinforcement learning abilities [24]. This task consisted of 220 trials presented in Matlab. Participants were instructed to select one of two boxes, and to try and judge which box had the highest probability of producing a reward (probability changed trial by trial according to Gaussian random walk). The outcome measure was the percentage of trials where the patient selected the box with the highest probability (optimal choice).

13) A non-verbal version of the Sustained Attention to Response Task (SART) [25]: this is a go/no-go task presented in E-Prime. In each trial one of two pictures of different men is displayed on the screen (one man represents the 'go' trial and the other represents is the 'no go' trial). Participants were instructed to press a button each time the go picture was shown, but to withhold their response when the no-go picture was displayed. There were 191 go trials and 24 no-go trials. Five measures are derived from this test: accuracy; errors of omission (failing to press on a 'go' trial); errors of commission (pressing on a 'no-go' trial); post-error slowing; and reaction times to correct 'go' trials.

14) Brixton spatial anticipation test [26]: This test assesses executive functions including reasoning, anticipation, cognitive control, solving problems, and cognitive flexibility. This task consists of 55 trials using the same template formed of ten circles with one coloured blue. Participants were instructed to point to where the blue position would be in the next trial, according to a pattern. The outcome measure was the number of errors and then transformed in a scaled score.

15) Visual short-term memory task: this task was created to test visual short-term and visual working memory. It consists of 14 trials presented in E-prime. Participants saw five grey squares located horizontally. In each trial, some of the squares were lit up in a particular order. Participants were instructed to remember and reproduce the sequence by button press. The outcome measure was the total number of sequences correctly reproduced (maximum score= 14).

16) 4-Way Weigl [27]: this is an alternative version of the WCST used to test executive functions including solving problems, cognitive flexibility, behaviour to achieving a goal, and response inhibition. Participants were presented with 12 coloured plastic tokens. They were instructed to sort the tokens in one of up to four options (colour, shape, symbol and texture). The outcome measure was total score (maximum score= 12). Moreover, secondary variables were also calculated: 1) the number of failures to complete the sort (more than 2 tokens are left unsorted); and 2) Perseveration type A was the repetition of a

previous sort. Perseveration type B involved the interruption of a correct sort to reverse the tokens to a previous sort.

7. Details of the Analyses

Our explanatory (in-sample) analyses involve deriving multivariable models which best explain the patients' responses to treatment. We use the Automatic Linear Modelling facility (ALM) distributed within the SPSS software package to build these models. The ALM facilities implements a stepwise, forward feature selection process: starting with an empty model, the process adds the single predictor whose addition most optimises (minimises) the resulting model's Akaike Information Criterion (AIC). The process then proceeds in iterations, greedily optimising the model by adding the best new predictor to those already selected, until no new feature's addition confers improvement greater than a penalty for increasing model complexity [28].

We repeat this process three times: (a) with only the 28 pre-treatment behavioural and 4 demographic variables; (b) with only the 69 lesion location variables, extracted from structural MRI and encoded using anatomically defined regions of interest (as described in section 5); and (c) with all of the data together. Each analysis yields an AIC value, and while these values are not meaningful in themselves, the differences between them can be expressed as Bayes Factors, which quantify the relative evidence that one model whose AIC value is better (lower) than another, is in fact the better model. Specifically, Bayes Factor = exp ((AIC1 - AIC2) / 2), where AIC2 is the smaller (better) of the pair. Bayes Factors >150 imply 'very strong' evidence in favour of the better model [29].

Our predictive analysis embeds the explanatory analysis within a leave-one-out cross-validation. This analysis proceeds in folds; in each fold, a single 'test' patient is removed from the sample, and the remaining 'training' patients are used to train a multivariable model defined using the SPSS ALM, as before. In this case, we also employ boosting within each fold, which creates a series of N models rather than a single model, in each fold of the analysis: here, we use N=10, which is the default setting in SPSS. Boosting produces a succession of "component models", each of which is built on the entire dataset. Prior to building each successive component model, the records are weighted based on the previous component model's residuals. Cases with large residuals are given relatively higher analysis weights so that the next component model will focus on predicting these records well. Together these component models form an ensemble model. The ensemble model scores new records by taking the mean prediction from the component models.

8. Analyses of change at longer-term follow-up

Our main analysis is focused on change observed immediately post-treatment, but therapy effects are usually not consistently maintained over time after the therapy ends[30]. This begs the question of whether: (a) longer-term therapy effects are best explained by the same pre-treatment variables which best explained the immediate treatment responses; and (b) longer-term therapy effects are also predictable from pre-treatment data.

We cannot answer the first question directly, because 2 of our original 23 patients dropped out of the study before their longer-term treatment effects could be measured (~3 months post-treatment). Nevertheless, we did test whether a model including all of the variables in our best (combined data) model for the immediate treatment responses, could also explain those later therapy effects. When fitted to the follow-up data, this model has an AIC of 78.0 (adjusted $R^2 = 0.18$): this result cannot be directly compared to that in the main text, because each refers to different patients and data, but our best model for treatment responses immediately post-treatment clearly does not capture longer-term treatment effects as well.

However, those later responses do still appear to be predictable, as assessed following the method used in our original predictive analysis: r (predicted response, empirical response) = 0.55, $p = 0.009$. Predictions made using models driven only by: (a) pre-treatment behavioural and demographic data; or (b) neuroimaging data; were not significantly correlated with empirical responses ($r = 0.40$, $p = 0.08$, and $r=0.22$, $p = 0.35$, respectively), though this was marginal for the 'behaviour + demographics' analysis. In any case, this underlines our central point that individual patients' therapy responses are in principle predictable, from pre-treatment data alone, and that the combination of pre-treatment behavioural data, demographic data, and lesion location data, is required to make those predictions well.

Supplementary References

1. Brysbaert M, New B. Moving beyond Kucera and Francis: a critical evaluation of current word frequency norms and the introduction of a new and improved word frequency measure for American English. Behavior research methods 2009;**41**(4):977-90.
2. Leff A, Starrfelt R. *Alexia: diagnosis, treatment and theory*: Springer Science & Business Media, 2013.
3. Plaut DC, McClelland JL, Seidenberg MS, et al. Understanding normal and impaired word reading: computational principles in quasi-regular domains. Psychological review 1996;**103**(1):56.
4. Crisp J, Howard D, Lambon Ralph MA. More evidence for a continuum between phonological and deep dyslexia: Novel data from three measures of direct orthography-to-phonology translation. Aphasiology 2011;**25**(5):615-41.
5. Woollams AM, Ralph MAL, Plaut DC, et al. SD-squared: on the association between semantic dementia and surface dyslexia. Psychological review 2007;**114**(2):316.
6. Woodhead ZVJ, Penny W, Barnes GR, et al. Reading therapy strengthens top–down connectivity in patients with pure alexia. Brain : a journal of neurology 2013;**136**(8):2579-91.
7. Callaghan MF, Josephs O, Herbst M, et al. An evaluation of prospective motion correction (PMC) for high resolution quantitative MRI. Frontiers in Neuroscience 2015;**9**:97.
8. Helms G, Dathe H, Dechent P. Quantitative FLASH MRI at 3T using a rational approximation of the Ernst equation. Magnetic resonance in medicine 2008;**59**(3):667-72.
9. Weiskopf N, Suckling J, Williams G, et al. Quantitative multi-parameter mapping of R1, PD(*), MT, and R2(*) at 3T: a multi-center validation. Frontiers in Neuroscience 2013;**7**:95.
10. Ashburner J, Friston KJ. Unified segmentation. NeuroImage 2005;**26**(3):839-51.
11. Seghier ML, Ramlackhansingh A, Crinion J, et al. Lesion identification using unified segmentation-normalisation models and fuzzy clustering. NeuroImage 2008;**41**(4):1253-66.
12. Oishi KF, A. F.; van Zijl P. C. M.; Mori, S. *MRI Atlas of Human White Matter*, 2011.
13. Hua K, Zhang J, Wakana S, et al. Tract probability maps in stereotaxic spaces: analyses of white matter anatomy and tract-specific quantification. NeuroImage 2008;**39**(1):336-47.
14. Tzourio-Mazoyer N, Landeau B, Papathanassiou D, et al. Automated Anatomical Labeling of Activations in SPM Using a Macroscopic Anatomical Parcellation of the MNI MRI Single-Subject Brain. NeuroImage 2002;**15**(1):273-89.
15. Eickhoff SB, Stephan KE, Mohlberg H, et al. A new SPM toolbox for combining probabilistic cytoarchitectonic maps and functional imaging data. NeuroImage 2005;**25**(4):1325-35.
16. Keuleers E, Brysbaert M. Wuggy: A multilingual pseudoword generator. Behavior research methods 2010;**42**(3):627-33.
17. Neale MD. *Neale analysis of reading ability: Reader*: ERIC, 1999.
18. Chue WL, Rose ML, Swinburn K. The reliability of the Communication Disability Profile: A patient-reported outcome measure for aphasia. Aphasiology 2010;**24**(6-8):940-56.
19. Swinburn K, Porter, G., and Howard, D. *Comprehensive Aphasia Test*: Psychology Press, 2004.
20. Wechsler D. Wechsler adult intelligence scale–Fourth Edition (WAIS–IV). San Antonio, Texas: Psychological Corporation 2014.
21. Robson H, Keidel JL, Ralph MA, et al. Revealing and quantifying the impaired phonological analysis underpinning impaired comprehension in Wernicke's aphasia. Neuropsychologia 2012;**50**(2):276-88.
22. Howard D, Patterson KE. *The Pyramids and Palm Trees Test: A test of semantic access from words and pictures*: Thames Valley Test Company, 1992.

23. Cattell RB, Cattell A. *Cattell Culture Fair Intelligence Test: A Measure of "g"*: Bobbs-Merrill, 1960.
24. Chowdhury R, Guitart-Masip M, Lambert C, et al. Dopamine restores reward prediction errors in old age. Nature neuroscience 2013;**16**(5):648.
25. Manly T, Davison B, Heutink J, et al. Not enough time or not enough attention? Speed, error and self-maintained control in the Sustained Attention to Response Test (SART). Clinical Neuropsychological Assessment 2000;**3**(10).
26. Burgess P, Shallice T. The Hayling and Brixton tests—Manual. London: Thames Valley Test Company: Harcourt Assessment, 1997.
27. Beglinger LJ, Unverzagt FW, Beristain X, et al. An updated version of the Weigl discriminates adults with dementia from those with mild impairment and healthy controls. Archives of clinical neuropsychology : the official journal of the National Academy of Neuropsychologists 2008;**23**(2):149-56.
28. Akaike H. Akaike's Information Criterion. In: Lovric M, ed. International Encyclopedia of Statistical Science. Berlin, Heidelberg: Springer Berlin Heidelberg, 2011:25-25.
29. Kass RE, Raftery AE. Bayes Factors. Journal of the American Statistical Association 1995;**90**(430):773-95.
30. Beeson PM, Robey RR. Evaluating single-subject treatment research: Lessons learned from the aphasia literature. Neuropsychology review 2006;**16**(4):161-69.
